# Supplementary material for: Single microwave-photon detector using an artificial Λ-type three-level system
Source: Nat Commun. 2016 Jul 25;7:12303. doi: 10.1038/ncomms12303 (PMC4962486; doi:10.1038/ncomms12303)
Supplement: Supplementary Information — Supplementary Figures 1-5, Supplementary Notes 1-5 and Supplementary References. [file ncomms12303-s1.pdf]

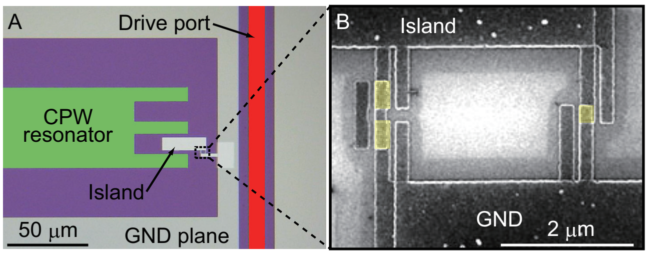

Supplementary Figure 1: Image of the qubit-resonator coupled system. **A,** False-colored optical image of the device magnified at the qubit part. The qubit (white) is coupled to the center conductor of the coplanar waveguide (CPW) resonator (green) through a capacitance of 4 fF. **B,** Scanning electron micrograph of the three-junction flux qubit. The areas shaded by yellow indicate the Josephson junctions.

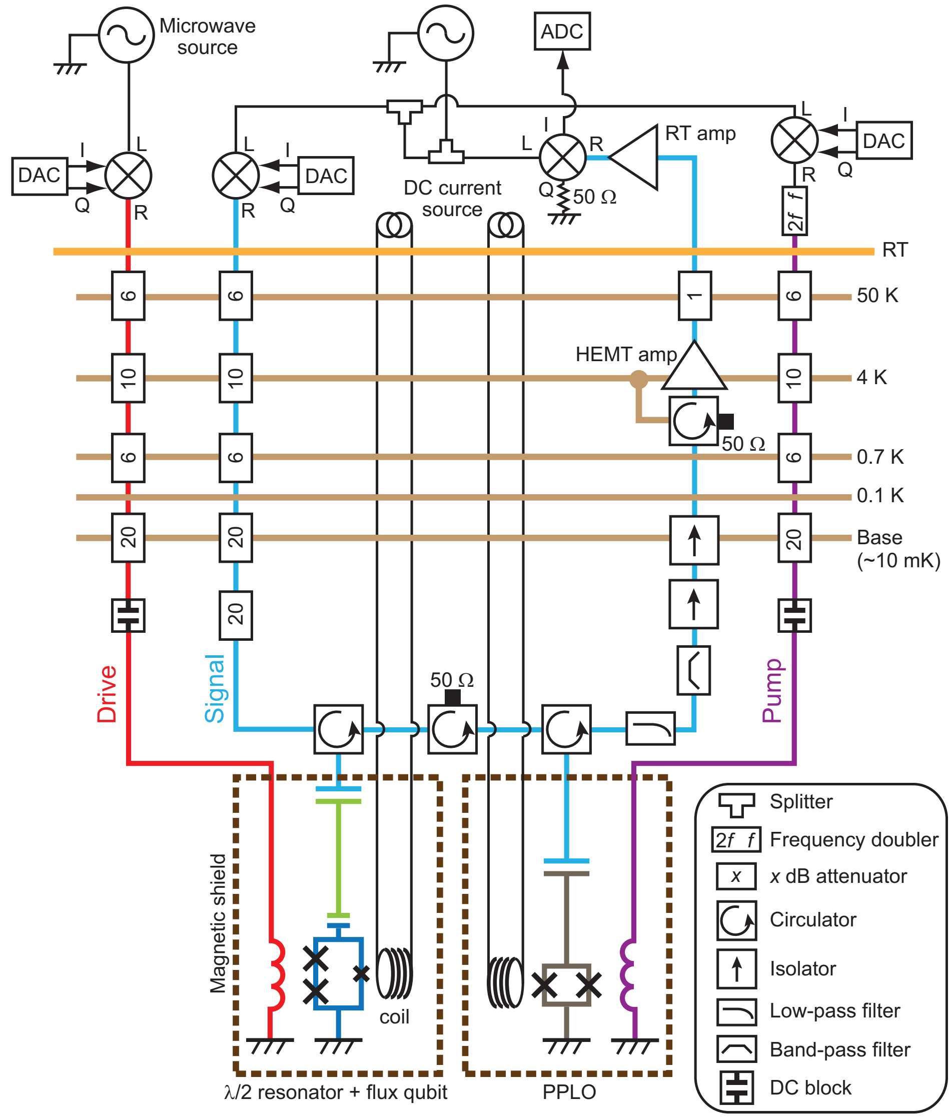

Supplementary Figure 2: Experimental setup diagram.

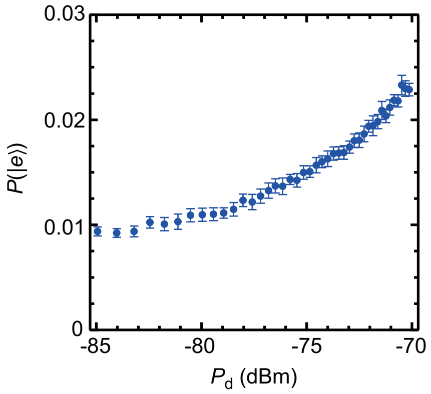

Supplementary Figure 3: Dark count probability in the detector. The data was taken ten times each and averaged before and after the measurement in Fig. 2b. The dark count probability including the imperfect initialization shows $0.014 \pm\ 0.001$ at $P_{\rm d}\ =\ -75.5$ dBm where the single-photon-detection efficiency hits the maximum. The error bars represent the standard deviation in twenty identical measurements.

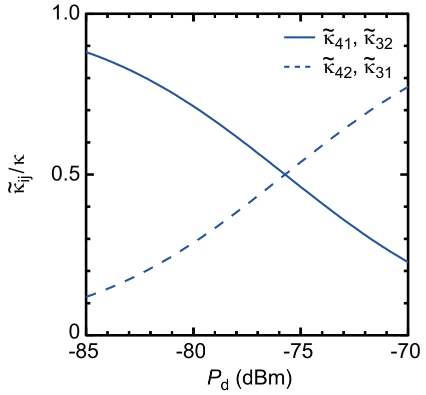

Supplementary Figure 4: Radiative decay rates of the impedance-matched Λ system, which are calculated based on the experimental parameters, as a function of the drive power. The two relevant decay rates, $\tilde{\kappa}_{41}$ and $\tilde{\kappa}_{42}$ or $\tilde{\kappa}_{31}$ and $\tilde{\kappa}_{32}$, become identical at $P_{\rm d} = -75.7$ dBm, where the impedance matching takes place.

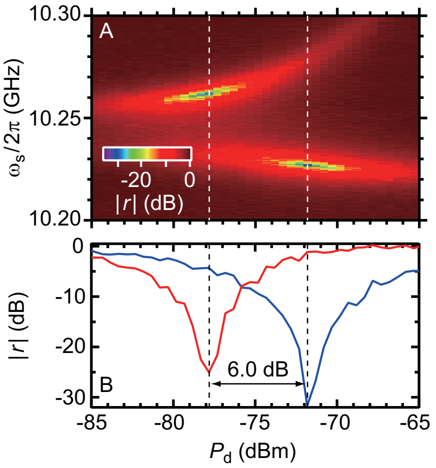

Supplementary Figure 5: Impedance-matched points. **A,** Amplitude of the reflection coefficient $|r|$ of a continuous input signal as a function of its frequency $\omega_{\rm s}$ and the drive power $P_{\rm d}$. Two dips corresponding to absorptions of the input microwave due to the impedance matching are observed. **B,** Cross-sections of A at $\omega_{\rm s}/2\pi = 10.227$ GHz (blue curve) and 10.262 GHz (red curve). Difference in $P_{\rm d}$ between two dips is $P_{\rm diff} = 6.0$ dB.

Supplementary Note 1: Device

Our device is composed of a $\lambda/2$ superconducting coplanar waveguide (CPW) resonator and a superconducting flux qubit (Fig. 1a). The CPW resonator is made of a 50-nm-thick Nb film sputtered on a 300-μm-thick undoped silicon wafer with a 300-nm-thick thermal oxide on the surface. It is patterned by electron-beam (EB) lithography using the ZEP520A-7 resist and $\mathrm{CF_4}$ reactive ion etching. The flux qubit with three Josephson junctions, where one is made smaller than the other two by a factor of α, is fabricated by EB lithography and double-angle evaporation of Al using PMMA (50 nm)/Ge (50 nm)/MMA (400 nm) trilayer resist (Supplementary Fig. 1B). The thicknesses of the bottom and the top Al layers separated by an $\mathrm{Al_2O_3}$ layer are 20 and 30 nm, respectively. In order to realize a superconducting contact between Nb and Al, the surface of Nb is cleaned by Ar ion milling before the evaporation of Al. The qubit is located at one end of the resonator and is coupled to the resonator dispersively through a capacitance of 4 fF, while it is coupled to the drive port inductively (Supplementary Fig. 1A).

The flux qubit is always biased with a half flux quantum where the transition frequency of the qubit ω_{ge} from the ground state $|g\rangle$ to the excited state $|e\rangle$ is $2\pi \times 5.508$ GHz ($T_1 \sim 700$ ns during photon-detection experiments), while the resonator frequency ω_{r} is $2\pi \times 10.256$ GHz (Q factor ~ 630) when the qubit is in the $|g\rangle$ state. It is shifted by a dispersive interaction with the qubit of $-2\chi = -2\pi \times 69$ MHz, which is enhanced by the straddling effect (*1*) and the capacitive coupling (*2*) when the qubit is in the $|e\rangle$ state. Note that ω_{ge} and ω_{r} denote not their bare frequencies but the renormalized ones including the dispersive shifts (*3*).

A parametric phase-locked oscillator (PPLO) (*4*), which is previously operated as a flux-driven Josephson parametric amplifier (JPA) (*5*) consists of a $\lambda/4$ superconducting CPW resonator terminated by a dc-SQUID (superconducting quantum interference device). A pump port is coupled to the SQUID loop inductively. The device was fabricated by the planarized Nb trilayer process at MIT Lincoln Laboratory. The resonator and the pump port are made out of a 150-nm-thick Nb film sputtered on a Si substrate covered by a 500-nm-thick $\mathrm{SiO_2}$ layer. A static resonant frequency of the PPLO is $\omega_{\mathrm{r}}^{\mathrm{PO}} = 2\pi \times 10.948$ GHz. The PPLO chip is the same as the one used in Ref. (*4*).

Supplementary Note 2: Experimental setup

A schematic of the measurement setup including the wiring in a cryogen-free ^{3}He/^{4}He dilution refrigerator, circuit components, and instruments used in the experiment is shown in Supplementary Fig. 2.

The qubit+resonator and the PPLO circuits are fabricated on separate chips and are separately mounted in microwave-tight packages equipped with an independent coil for the flux

bias. They are protected independently by the Cryoperm magnetic shield from an external flux noise such as the geomagnetic field.

Microwave pulses for the drive, signal, and pump ports are generated by mixing the continuous microwaves with pulses which have independent IF frequencies generated by DACs (digital to analog converter) developed by Martinis group at UCSB (*6*). The pulses are applied through the input microwave semi-rigid cables, each with attenuators of 42 dB in total, and DC-blocks for the drive and pump ports. For the signal port, the microwave pulses are further attenuated by 20 dB, and are input to the resonator through a circulator to separate the input and reflected waves. The reflected waves are routed to PPLO via three circulators (9-11 GHz) and are reflected there again, and are propagated through a low-pass ($f_c = 12.4$ GHz) and band-pass filters (9-11 GHz), two isolators (9-11 GHz), and the circulator (9-11 GHz) with a $50\ \Omega$ termination. Finally, the signals are amplified by a cryogenic HEMT amplifier and a room-temperature amplifier with a total gain of ~ 66 dB, and mixed with a local oscillator at an I/Q mixer down to the IF frequency. The I component of the reflected signals are sampled at 1 GHz/s by an ADC (analog to digital converter).

For the impedance-matching measurement (Fig. 2a), the PPLO is kept off. Namely, pump pulse is off (the output from the DAC in the pump port is turned off) and $\omega_{\mathrm{r}}^{\mathrm{PO}}$ is far detuned from ω_{r} so that the PPLO acts as a perfect mirror. In other measurements, the PPLO is kept on.

Supplementary Note 3: Dark count in the detector

Supplementary Fig. 3 shows the dark count probability in the detector, which is the click probability without applying the signal pulse in the pulse sequence of Fig. 1c. The dark count is mainly caused by the nonadiabatic qubit excitation due to the drive pulse and the imperfect initialization. The probability induced by the latter factor is constant and is measured to be 0.008 ± 0.001, while the probability induced by the former factor depends on the power and the length of the drive pulse and remains finite even with the Gaussian envelope. We determine the dark count probability before and after each measurement of Figs. 2b, 2e, and Fig. 3 and subtracted the averaged value from the measurement result.

Supplementary Note 4: Time constant of an impedance-matched Λ system

We denote the overall decay rate of the resonator by κ and the radiative decay rate for the $|\tilde{i}\rangle \rightarrow |\tilde{j}\rangle$ transition in the Λ system by $\tilde{\kappa}_{\mathrm{ij}}$. Supplementary Fig. 4 shows $\tilde{\kappa}_{\mathrm{ij}}/\kappa$ as a function of the drive power P_{d}, calculated based on the experimental parameters. In the experiment, we choose $P_{\mathrm{d}} = -75.5$ dBm where the photon detection efficiency η reaches the maximum. At this point, $\tilde{\kappa}_{41}/\kappa = 0.49$. The time constant of the impedance-matched Λ system for the voltage

amplitude decay, τ_Λ, is estimated to be $2/\kappa \sim 20$ ns, where $\kappa = \tilde{\kappa}_{41} + \tilde{\kappa}_{42} \sim 2\pi \times 16$ MHz. The shortest signal pulse length is 34 ns in Fig. 3, which is comparable with τ_Λ.

Supplementary Note 5: Input-power calibration

To estimate the photon detection efficiency precisely, calibration of the signal microwave power level on the sample chip is required. For the calibration, we measure the reflection coefficient as a function of the signal microwave frequency ω_s and the drive power P_d and determine the impedance-matched points (Supplementary Fig. 5). Here, we use continuous microwaves for both the signal and the qubit drive, and set the drive frequency at $\omega_\mathrm{d} = \omega_\mathrm{ge} - 2\pi \times 46$ MHz. We observe two dips representing the impedance matching, similarly to the inset of Fig. 2a. In the limit of weak signal power and no intrinsic loss of the resonator, these dips are expected to appear at the same P_d, where the two radiative decay rates of the Λ system are balanced, $\tilde{\kappa}_{31} = \tilde{\kappa}_{32}$ and $\tilde{\kappa}_{41} = \tilde{\kappa}_{42}$ (*7*), where $\tilde{\kappa}_\mathrm{ij}$ is the radiative decay rate for the $|\tilde{i}\rangle \rightarrow |\tilde{j}\rangle$ transition in the impedance-matched Λ system. In the actual system, however, the finite population in the level $|\tilde{2}\rangle$ as well as the intrinsic loss of the resonator weakens the elastic photon scattering from the Λ system, and the impedance matching occurs when the radiative decay rates are not balanced, $\tilde{\kappa}_{31} > \tilde{\kappa}_{32}$ and $\tilde{\kappa}_{41} > \tilde{\kappa}_{42}$ (*3*). This yields a difference in the drive power, P_diff, between the two dips. P_diff is sensitive to the input signal power: As we increase the signal power, the level $|\tilde{2}\rangle$ is more populated and P_diff gets larger. Note that the small P_diff observed in the inset of Fig. 2a is attributed to the intrinsic loss of the resonator, since the pulsed signal field is sufficiently weak in this measurement.

We use P_diff to calibrate the signal power level. We determine the signal power level which reproduces $P_\mathrm{diff} = 6.0$ dB (Supplementary Fig. 5B) by the numerical simulation, following Ref. (*7*). In the numerical simulation, we employ the following parameters which are estimated by independent measurements: the qubit decay rate $\Gamma/2\pi = 0.174 \pm 0.012$ MHz (during this measurement T_1 shows $\Gamma^{-1} = 919 \pm 62$ ns) and the ratio of the external and total decay rates of the resonator photon $\kappa_\mathrm{ext}/\kappa = 0.964 \pm 0.003$ (for other parameters, see "Device" section of this supplementary material). As a result, the signal power is estimated to be $P_\mathrm{s} = -145.28$ dBm at maximum ($\Gamma/2\pi = 0.186$ MHz and $\kappa_\mathrm{ext}/\kappa = 0.967$) and $P_\mathrm{s} = -146.02$ dBm at minimum ($\Gamma/2\pi = 0.162$ MHz and $\kappa_\mathrm{ext}/\kappa = 0.961$). Therefore, $P_\mathrm{s} = -145.65 \pm 0.37$ dBm. This agrees well with an independent estimation of $P_\mathrm{s} = -146.0$ dBm by taking into account the total losses in the input port.

Supplementary References

1. Koch, J. *et al.*, Charge-insensitive qubit design derived from the Cooper pair box. *Phys. Rev. A* **76**, 042319 (2007).

2. Inomata, K., Yamamoto, T., Billangeon, P.-M., Nakamura, Y. & Tsai, J. S. Large dispersive shift of cavity resonance induced by a superconducting flux qubit in the straddling regime. *Phys. Rev. B* **86**, 140508(R) (2012).

3. Inomata, K. *et al.*, Microwave down-conversion with an impedance-matched Λ system in driven circuit QED. *Phys. Rev. Lett.* **113**, 063604 (2014).

4. Lin, Z. R. *et al.*, Josephson parametric phase-locked oscillator and its application to dispersive readout of superconducting qubits. *Nat. Commun.* **5**, 4480 (2014).

5. Yamamoto, T. *et al.*, Flux-driven Josephson parametric amplifier. *Appl. Phys. Lett.* **93**, 042510 (2008).

6. URL : http : //web.physics.ucsb.edu/ martinisgroup/electronics.shtml.

7. Koshino, K., Inomata, K., Yamamoto, T. & Nakamura, Y. Implementation of an impedance-matched Λ system by dressed-state engineering. *Phys. Rev. Lett.* **111**, 153601 (2013).
